# Determinants of research productivity during postgraduate medical education: a structured review

**DOI:** 10.1186/s12909-021-03010-1

**Published:** 2021-11-09

**Authors:** Kevin B. Laupland, Felicity Edwards, Jayesh Dhanani

**Affiliations:** 1 Department of Intensive Care Services, Royal Brisbane and Women’s Hospital, Level 3 Ned Hanlon Building, Butterfield Street, Brisbane, Queensland 4029 Australia; 2 Queensland University of Technology (QUT), Brisbane, Queensland Australia; 3 Faculty of Medicine, University of Queensland, Brisbane, Queensland Australia

**Keywords:** Medical training, Research productivity, Postgraduate, Trainees

## Abstract

**Background:**

Although formal participation in research is an integral and often mandatory component of clinical training programs, resulting productivity is highly variable. The objective of this review was to identify determinants of successful research performance among graduate medical education trainees.

**Methods:**

A structured review of the published literature was performed by searching PubMed, CINAHL, and EMBASE from inception through to 7 April, 2021. Articles examining graduate medical education trainee research productivity evidenced by publications in peer-reviewed journals were included.

**Results:**

Eighty-five articles were included of which most (66; 78%) were reported from the USA or Canada (10; 12%). A wide range of disciplines were represented with the most common being general surgery, internal medicine, orthopedic surgery, and pediatrics. Themes (number of reports) included trainee characteristics (*n* = 24), project characteristics (*n* = 8), mentoring/supervision (*n* = 11), and programmatic aspects (*n* = 57). Although variable results were observed, research productivity tended to be higher with prior research experience, later years of training, male gender, and pursuit of a postgraduate degree. Few project related aspects of success were identified. Trainee publication was associated with mentors with higher rank, publication productivity, and supportive academic environments. Training programs with organised programs/curricula including protection of time for research were associated with increased productivity as were provision of incentives or rewards but not mandatory requirements.

**Conclusion:**

This review identifies several trainee characteristics, project and mentor aspects, and programmatic aspects associated with increased productivity that may serve as a useful resource for trainees and graduate medical education training programs.

**Supplementary Information:**

The online version contains supplementary material available at 10.1186/s12909-021-03010-1.

## Introduction

Research is recognized as an important component of graduate medical education training, and active participation is mandatory in many programs globally [1, 2]. Trainees may benefit by participation in research through an improved undertanding of and ability to apply studies reported in the literature, which may in turn translate to better performance on clinical examinations and patient outcomes [3]. In addition, research productivity during graduate medical education has been shown in many disciplines to increase the likelihood of acceptance into advanced training positions and predicts success in subsequent academic careers [4, 5]. Although research is a common requirement, a minority of graduate medical trainees publish their work in peer-reviewed journals, and this outcome is highly variable among individuals, disciplines, and institutions [6].

Knowledge of the determinants of successful research performance during graduate medical education is important for both individuals and for training programs. Although systematic reviews summarizing factors associated with successful performance of graduate medical education research have been reported, they have either been limited to interventions at the programmatic level, highly selected jurisdictions or interventions, or have included non-research related scholarly activities [7–10]. The objective of this study was to conduct a structured review of the literature to broadly identify factors associated with research productivity during graduate medical education to serve as a resource for both clinical trainees and program directors.

## Methods

The study protocol was established a priori and was developed as outlined by the members of the Joanna Briggs Institute and members of the Joanna Briggs Collaborating Centres [11].

The specific research questions were “what evidence is there to guide successful completion and publication of a graduate medical education trainee research project with respect to”:

1. Trainee background and characteristics;
2. Project characteristics;
3. Mentoring/supervision; and
4. Programmatic aspects.

### Eligibility criteria

Studies of any methodology that addressed one or more of the research questions were considered. Clinical studies were selected for inclusion if the primary focus was on physician graduate clinical trainees and the topic was conduct of novel research projects. Our focus was on clinical trainees who were enrolled in core training programs (i.e. internship, residency, registrar) following medical school with primary goal of granting of initial speciality designation and/or licensure. Research conducted by fellows of a (sub)specialty college post-certification were excluded in order to reduce the confounding effects of trainees who were licensed physicians/consultants who were pursuing advanced level research experiences. Program descriptions, quality improvement initiatives, or opinion surveys that did not include a comparative evaluation component were excluded as were case reports, reviews, editorials, or reports published only as abstracts. Our primary outcome was publication in a peer-reviewed journal.

### Search methodology

An initial electronic search of titles and abstracts was conducted using the Pubmed, EMBASE, and CINAHL databases from inception through to 7 April, 2021. The search used the terms “resident”[Title/Abstract] OR “registrar”[Title/Abstract] OR “trainees”[Title/Abstract]) AND “research”[Title]. The search was not limited by design, language, or year. The titles and abstracts were independently screened for potential inclusion by two reviewers (KBL, JD) with consensus review of discrepant results. Full length articles were then retrieved and reviewed by one author (KBL) with application of eligibility criteria. Additional relevant publications were identified by scanning of bibliographies of included articles and review articles [7–10].

Following the compilation of a list of articles for inclusion, data was extracted with results mapped according to the pre-specified themes. Studies were classified as pre-post intervention (i.e. historical control cohort), cohort (observational group or series with subsequent outcome over a time period), survey/cross sectional (performed at a defined time point), and other. Where other outcome measures were bundled with publications we limited data inclusion to publications only where data were available. Where there was inadequate data to analyse publications separately we only included studies reporting a composite outcome measure where publication was a major component.

Analysis was descriptive. Study results were grouped into themes according to the research questions. We accepted and reported statistical significance of individual comparisons from the original studies without secondary or confirmatory analysis and did not calculate summary statistics or perform meta-analysis. Statistical significance was defined by a *p*-value of ≤0.05.

## Results

**Fig. 1:**
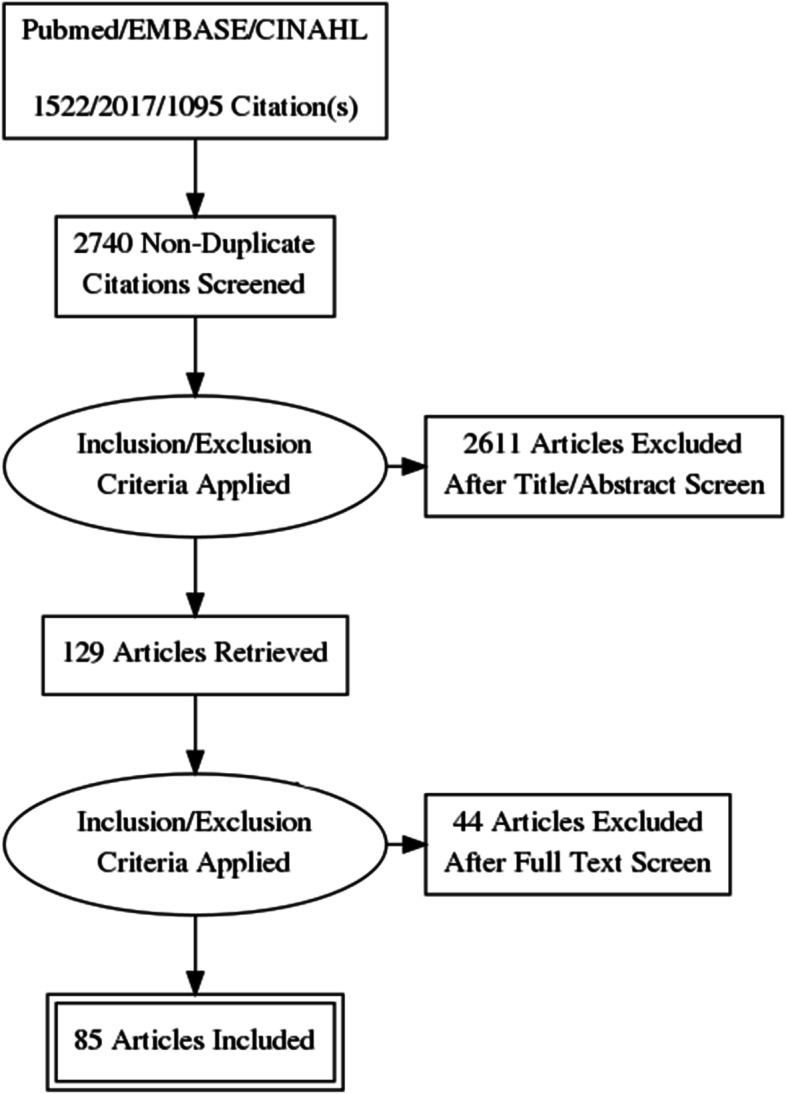
PRISMA diagram of study selection

Electronic searches conducted using Pubmed (*n* = 1522), EMBASE (*n* = 2017), and CINAHL (*n* = 1095) databases retrieved a total of 4634 citations of which 2703 remained following duplicate removal. An additional 37 citations were identified from bibliographic review of references of included articles and 129 full text articles were retrieved for full text review. After application of study inclusion and exclusion criteria, 85 articles were included in the final review as detailed in Fig. 1. The details of each of the studies are included in supplementary Table 1.

Of the 85 included studies, 66 (78%) were reported from the USA, with ten (12%) from Canada, two (2%) from Thailand, and one (1%) each from Australia/New Zealand, USA/Canada, Europe, Germany, India, Japan, and Lebanon. Study designs were pre-post (40; 47%), cohort (23; 27%), survey/cross sectional (21; 25%), and one study was a mixed methods (pre-post with cohort). Among the 77 studies where the number of subjects were reported or could be estimated from the manuscript, the median number was 115 with a range from 14 to 1690. A wide range of disciplines were represented with the most common being general surgery, internal medicine, orthopedic surgery, and pediatrics as shown in Table 1. Studies overall included subjects from as early as 1965 to as late as 2019, and the median start and end dates were 2004 and 2012, respectively.

**Table 1:** Distribution of training disciplines of included studies

Discipline	Number (%)
Internal Medicine	11 (13%)
Surgery	
General	11 (13%)
Orthopedic	8 (9%)
Urology	6 (7%)
Otolaryngology	4 (5%)
Plastic	3 (4%)
Neurosurgery	1 (1%)
Obstetrics and Gynecology	1 (1%)
Other	2 (2%)
Pediatrics	8 (9%)
Radiation Oncology	6 (7%)
Family Medicine	5 (6%)
Psychiatry	5 (6%)
Anesthesia	3 (4%)
Emergency Medicine	3 (4%)
Physical Medicine and Rehabilitation	3 (4%)
Neurology	2 (2%)
Psychosomatic and General Internal Medicine	1 (1%)
Radiology	1 (1%)
Other	1 (1%)

### Trainee background and characteristics

Twenty-four studies included examination of aspects related to trainee attributes or characteristics that were associated with research productivity [12–35], and these were largely related to past research performance, clinical training experience, and gender.

Six studies examined prior publication record related to past research experience [12, 13, 22, 31, 34, 35], of which three found this to be associated with increased productivity [12, 34, 35]. One study also observed that domestic medical graduates were more likely to publish than those who attended foreign medical schools [12]. There were mixed effects associated with trainees who had prior or concomitant pursuit of a higher postgraduate degree (i.e. MSc, MPH, PhD). Among a total of 13 studies, this was associated with higher productivity in six [15, 23–26, 34], no difference in five [13, 21, 28, 31, 33], and in two studies this was associated with lower productivity [18, 19]. Of four studies that looked at past research experience that was not specifically related to higher degree [12, 26, 29, 32], three found this to be a significant factor associated with graduate medical training research productivity [12, 26, 32].

Clinical training experiences were associated with productivity. Most studies found that later years of training [14, 17, 23, 29, 31, 32] were associated with higher publication rates with no difference observed in three studies [12, 21, 27], and an inverse effect in one [22]. Notably, one study found that residents who reported doing a higher number of histories and physicals per week had higher research productivity [26]. Trainees who reported an expressed interest in doing research were more productive in three [16, 23, 29] studies with only one showing no difference with this variable [33].

Eleven studies examined the gender of trainees as a determinant of publication success and found that males had higher productivity in five studies [18–20, 29, 32], females in one study [26], and no difference was observed in five studies [12, 13, 17, 21, 30].

### Project characteristics

Eight studies examined aspects of specific projects in relation to success with subsequent publication [12, 16, 21, 36–40]. Although specific project supports played a role, the main findings were related to the choice of study designs and subsequent publication success.

Vinci et al examined factors associated with a successful research productivity (publication or presentation) among pediatric trainees and found that this was significantly related to the project type [21]. While success occurred with 38% of educational/curriculum, 54% of clinical, and 57% of basic science projects, only 6% of “enhanced clinical experience” projects, and no career planning projects were successfully published. On the other hand, Atreya and colleagues examined 94 studies conducted by American Internal Medicine trainees of which 32% were retrospective cohorts, 44% were cross sectional, 4% were prospective cohorts, and 20% were other designs [12]. However, there was no significant association of research productivity with study design [12]. Yumeen and colleagues reported that among projects presented at a Plastic Surgery research day, subsequent publications rates were higher for basic science (9/14; 64%) as compared to clinical (42/113; 37%) topics [40].

Taschanchai and Mahachoklertwattana found that availability of funding influenced the types of successful studies published during Pediatrics residency in Thailand, with increasing funds associated with fewer retrospective studies and increasing cross sectional and prospective studies [38]. Winn et al. found no difference in publication rates among residents who received project funding grants or not in an American Pediatrics program [39]. Among three studies that looked at the availability of a research assistant, two showed a positive effect [16, 36] with no effect in one [12]. The latter study also found that biostatistical support was associated with publication [12] but this was not deemed to be of benefit in another study [37].

### Mentoring/supervision

Eleven studies examined mentor and/or supervisory characteristics in relation to subsequent success in publication of resident projects [6, 12, 21, 22, 41–47]. These mentoring aspects were specifically related to direct project supervision in some cases [12, 21, 22, 41, 42], whereas the mentoring environment was a consideration in others [6, 41, 43–48].

Atreya et al found that among Internal Medicine trainees, successful publication was significantly higher with a mentor who had an advanced degree (75% vs 50%), intramural funding (73% vs 52%), and five or more publications at study conception (96% vs 71%) [12]. However, external funding and protected time for the mentor were not associated with publications [12]. In a survey of Physical Medicine and Rehabilitation program directors in the USA, having a mentor external to the department was associated with a lower rate of publication [41]. Susarla et al found that Plastic Surgery mentor rank of associate/full professor versus lower ranks were associated with higher publication success [22]. Levitt et al found no significant effect of mentor financial support, research award, or protected time on publications productivity in Emergency Medicine training programs [42]. Vinci and colleagues reported that mentor ratings were associated with research productivity [21].

Aspects of the mentoring environment included availability of a mentor [43, 46], guidelines about mentor choice [41], and having a residency director with increased activity of publishing [44, 45] which were associated with increased publication output. Lepard et al surveyed neurosurgical programs in the USA and found that programs reporting journal clubs with mentors with epidemiology and biostatistics expertise, but not regular mentor meetings associated with increased resident publications [27]. Older, more established residency programs have been associated with improved publication outcomes among trainees [44]. Similarly, trainees in larger and/or tertiary care/university hospitals have demonstrated higher publication rates than those in smaller community-based hospitals [6, 46, 48]. One study of Family Medicine residents from Canada found that encouragement and support to publish finished projects resulted in increased publications [47].

### Programmatic aspects

These included programmatic/curricular aspects [23, 27, 36, 41, 46, 49–81], management of trainee time [13, 23, 26, 27, 41, 42, 46, 65, 74, 78, 82–92], mandatory requirements [16, 27, 41, 72, 86, 91–94] and rewards [42, 95, 96].

Most of the included literature surrounding aspects related to trainee publications were related to programmatic aspects, most commonly by implementation of a research program and/or curriculum with measure of publication output changes using pre-post designs. Among the 38 studies that evaluated multifaceted programs/curricula of varying types, most were associated with increased output [36, 46, 49–70, 80, 81], whereas 11 showed no significant effect [27, 41, 71–79], and one was associated with lower publication output [23].

Protection of trainee time for research found this to be a significant productivity factor in 12 studies [13, 23, 26, 27, 74, 82–88], whereas this was not significant in five [41, 42, 89–91]. Similarly, a specified/scheduled research block of research time or formalized research rotation increased productivity [46, 65, 78, 88, 92] in five studies whereas it was of no effect in two [36, 91]. Williams et al found that provision of research time in a longitudinal as compared to block or no time schedule was associated with a significant greater output of 1.9 ± 1.8 versus 1.0 ± 1.0 publication [85]. A dose response was found by Lee et al [82] with increasing residency research time from 0, 3–4, 6, and 12 months correlated with increased median residency publications of 1, 2, 3, and 5, respectively.

Institution of a mandatory research requirement had limited effect with only two [92, 93] of six papers finding a positive effect [16, 41, 72, 91–93]. Similarly, a mandatory manuscript/publication requirement increased publication in only one [93] of three studies [27, 93, 94]. Notably, in one study the requirement was associated with an adverse shift away from first author position [94]. Fisher found that an intervention whereby regular notification of research requirements and tracking of progress was associated with an increase in publications in an American surgical training program [86].

Rewards for productivity demonstrated significant benefit in two studies [95, 96]. Chang and colleages reported a pre-post study where a points based monetary reward system was implemented for academic productivity which led to a tripling of the average annual resident publication output [95]. Larsen examined the effect of a monetary incentive which although increased publications it was associated with a shift away from basic science projects and toward systematic reviews [96]. Research awards were not associated with increased publications in one survey of Emergency Medicine programs [42].

## Discussion

This report reviews graduate medical education trainee research productivity and identifies several determinants of outcome related to trainees, mentors, and programs. We identified a moderate sized body of literature of which most was related to programmatic aspects and very little specifically related to individual project characteristics. Individual and mentor factors are generally associated with experience, dedicated research time, and specific training. Implementation of research programs and/or curricula are broadly associated with improved publication outcomes and monetary incentives rather than mandatory requirements improves research productivity. These findings should be of value as a resource for individual trainees and to inform training programs in devising means to improve research productivity associated with graduate medical education.

Although there were mixed findings, generally speaking the literature indicates that trainees with prior research experience, previous or concomitant higher research degrees, and those at later years of training have increased research productivity. While the latter aspect may simply be a measure for increasing time of opportunity, greater prior experience(s) and past or concurrent pursuit of postgraduate degrees likely reflects increases in research related knowledge and aptitude. It is not surprising that trainee attitude towards research is associated with productivity [16, 23, 29]. It is a somewhat surprising finding that one study found that trainees on “busier” clinical services had higher research productivity despite presumably having less available time for activities not directly related to patient care [26]. The observation among included studies that males tend to have higher research productivity during graduate medical education is important. Further studies are needed in order to define whether this observation may be related to confounding factors (i.e. past experience, interest in research), systemic gender bias, and/or differential opportunity [97].

A limited body of literature evaluated project-specific and mentoring related aspects and research productivity. The small number of studies related to specific project types and aspects precludes general summary comments and identifies this as a priority for further investigation. With regards to mentorship, more experienced mentors with prior track record of productivity who are working within a research intensive department/environment are associated with supervision of trainee research publication. In recent decades many jurisdictions have re-distributed provision of medical education with less emphasis on large tertiary care urban settings with a shift to smaller communities. However, failure to concomitantly increase research mentors in these distributed centres presents a significant risk to trainee research productivity and merits further attention [98].

Most of the literature surrounding research productivity during graduate medical education has related to programmatic interventions and has been the focus of four previous systematic reviews [7–10]. It is important to note that while we identified more than 50 publications investigating this topic, most of the included studies relate to implementation of multi-faceted programs or bundled interventions such that individual variables related to outcomes are difficult to assess. In addition, this body of literature largely consists of studies employing pre-post historical cohort designs that have major inherent limitations. Prospective interventional studies are needed to best define ways to improve graduate medical education research productivity.

Although this review benefits from a structured approach, there are some limitations that merit discussion. We excluded conference proceedings and abstracts and as a result there may be additional relevant information that was not included. Additionally, our search strategy was simplistic. While we are confident that we identified relevant articles through our protocol including bibliographic review of included papers, it remains a possibility that we may have missed inclusion of some relevant articles. Another consideration is that abstraction of the results from the full text articles was conducted by one author raising the possibililty of error and/or bias in that regard. Ideally this would have been performed independently by more than one author and is a limitation of this report. It is notable that we did not formally evaluate statistical significance of individual studies nor try to calculate summary statistics [11]. Furthermore, we focussed on indexed publications as our primary outcome. While other scholarly activities have merit, publications in indexed journals represent the most widely accepted objective outcome of research productivity. Finally, while we included an assessment of gender, we did not specifically examine other aspects of potential discrimination (e.g. race) that could influence research productivity during training.

To conclude, this review details several trainee characteristics, mentor attributes, and programmatic aspects that are associated with increased graduate medical trainee research productivity. This information should be of value to both trainees and programs with their goals of improving research publication productivity during graduate medical education.

## Supplementary Information

**Additional file 1.**

## Data Availability

Not applicable.
