# Supplementary material for: Determinants of research productivity during postgraduate medical education: a structured review
Source: BMC Med Educ. 2021 Nov 9;21:567. doi: 10.1186/s12909-021-03010-1 (PMC8579624; doi:10.1186/s12909-021-03010-1)
Supplement: Supplementary file 1 — Additional file 1. [file 12909_2021_3010_MOESM1_ESM.docx]

**Supplementary Table 1**

| First author (reference) | Location and discipline | Design | Subjects/measures | Key findings |
| --- | --- | --- | --- | --- |
| Abramson [29] | Pediatrics, USA, 2016 | Survey | 464 (60%) of 771 residents | Scholarly activity including publications was associated with more senior trainee level, male gender, intention to do research after residency |
| Ahmad [36] | Anesthesia, USA, 2010/2011 | Survey | 100 (74%) of 131 program directors | 40% of programs with structured residency research programs had >20% trainees with publications versus 14% (p=0.01). Research rotations of 2 months or more no difference. Programs with >20% faculty involved in research associated with more resident publications (p=0.03) |
| Allen [71] | General Surgery, Canada, 2002-2012 | Pre-post | 68 | Non-significant increase in average number of publications per resident following implementation of a research program in 2007 (0.30 to 0.51; p=0.21) |
| Alweis [81] | Internal Medicine, USA, 2001-2015 | Pre-post | 27-42 per year | Increase in publications with iterative implementation of a multi-faceted program |
| Andrew [31] | Urology, Canada, 2011 | Survey | 42 (28%) of 152 | Publication output related to later years of training but not prior research experience or higher degree |
| Arora [50] | Psychiatry, USA, 2009-2019 | Pre-post | Not specified | Publication increase from 1 to 10 publications following implementation of research curriculum |
| Atreya [12] | Internal Medicine, USA, 2006-2013 | Cohort | 94 research projects | Utilization of epidemiology/biostats support (p<0.001), resident past research experience (p=0.04), USA medical education (p=0.05), and mentor advanced degree (p=0.02), publication record (p=0.001), and funding (p=0.05) associated with higher rate of publication |
| Blake [41] | Physiatry, USA, 1994 | Survey | 63 (87%) of 72 program directors | Significant factors associated with completed resident projects include mentors within department (p=0.03) and resident guidelines about choosing a mentor (p=0.046) |
| Carter [75] | Internal Medicine, USA, 2010-2015 | Pre-post | 115 | Non-significant increase in publications (26.9% vs 20.6%, p=0.43) after implementation of multifaceted program |
| Chan [23] | Orthopedics, Canada, 2005-2006 | Survey | 85 (45%) of 188 | Blocked research time (38% vs. 14%; p=0.012) and advanced degree (53% vs. 21%; p=0.011) |
| Chang [95] | Otolaryngology, USA, 1997-2011 | Pre-post | ≈140 (10-11 residents per year) | After implementation of a reward system, mean publication per resident per year increased from 0.13 to 0.43 (p=0.004) |
| Chen [13] | Otolaryngology, USA, 1996-2013 | Pre-post | 75 | Following work hour restrictions in 2003 mean number of publications per resident increased from 1.21 to 5.1 (p<0.001) |
| Crawford [44] | Family Medicine, USA, 2009 | Survey | 248 (55%) of 450 programs | Odds of programs having 25% or more residents publishing were residency director publishing (4.1; 95% CI, 1.5-11.5), 6 or more faculty publications within 2 years (7.8; 95% CI, 3.0-20.3), and residency established before 1980 (3.7; 95% CI, 1.4-9.6) |
| Desbiens [77] | Internal Medicine, USA, 1997-2006 | Pre-post | ≈90 (30 residents per year) | Implementation of a multifaceted program increased presentations but not evident increase in peer reviewed publications |
| Durning [45] | Internal Medicine, USA, 1992-2001 | Pre-post | 89 | Appointment of a resident research director was associated with an increase in publications from 6 to 17 (p=0.037) |
| Eckert [69] | Anesthesia, USA, 2007-2013 | Pre-post | 108 | Implementation of a multifaceted program increased publications from 0.31 to 0.52 per resident year |
| Farrokhyar [63] | Surgery, Canada, 2005-2013 | Pre-post | 207 | Implementation of a research curriculum was associated with increased residents publishing (68% vs 43%, p<0.001) |
| Fayad [14] | Postgraduate clinical trainees, Lebanon, 2015 | Survey | 290 | Publication rate was higher in trainees in later years of training but did not vary by speciality |
| Finkelstein  [87] | Urology, USA, 2009-2013 | Cohort | 148 | Residents with dedicated research year produced greater number of publications per year (1.2 vs 0.6; p=0.0092) than those who did not |
| Fisher [86] | General Surgery, USA, 2002-2005 | Pre-post | 16 | Programmatic compliance intervention associated with an increase publications from 3 to 13 |
| Frankel [51] | General Surgery, USA, 2010-2017 | Pre-post | 57 | Implementation of a multifaceted program was associated with increased output of average 2.3 to 8.5 publications per resident (p=0.01) |
| Gill [32] | Internal Medicine, Canada, 1993-1997 | Survey | 81 (66%) of 123 | Publication associated with males (RR 2.6; 95% CI, 1.1-6.1), 3^rd^ year or later level of training (RR2.1; 95% Ci, 1.3-3.2), prior research experience (RR 1.6; 95% CI, 1.0-2.5) |
| Grzbowski [47] | Family Medicine, Canada, 1990-1997 | Survey | 190 (71%) of 251 | Although only 7% of respondents published their projects, a sample of 7 of 15 projects were published after further encouragement |
| Gutovich [26] | Radiation Oncology, USA, 2010-2012 | Cohort | 97 (42%) of 232 | Research time most important determinant of publication productivity |
| Harrison [72] | General Surgery , USA, 2007-2017 | Pre-post | 388 | Implementation of a multifaceted program was associated with a non-significant increase in publications (22 to 35; p>0.05) |
| Hellenthal [34] | Urology, USA and Canada, 2007-2008 | Survey | 127 (50%) of 255 | Months of protected time, PhD before residency, and publication before residency associated with higher number of publications |
| Hepburn [68] | Internal Medicine, USA, 1990-1999 | Pre-post | ≈300 (30 residents per year) | Implementation of a multifaceted program increased publications from 3 to 21 |
| Holoyda [80] | Plastic Surgery, USA, 2012-2017 | Pre-post | 6-8 residents per year | Significant increase in publications (2 vs. 0.47 per year per resident; p=0.009) following introduction of quarterly research meetings |
| Hoedebecke [64] | Family Medicine, USA, 2011-2013 | Pre-post | Unclear number of residents | Implementation of a resident-led process improvement process led to increase of publications from 2 to 6 |
| Ishiguro [6] | Pediatrics, Japan, 2015-2016 | Survey | 1500 (%) of 1718 | Residents at community hospitals fewer peer-reviewed publications as compared to national/public university hospitals (odds ratio 0.53 (95% CI, 0.37–0.76) |
| Isom [15] | General Surgery, USA, 2000-2012 | Cohort | 511 | Higher median number of publications among 87 (17%) residents who obtained an advanced degree during training (9 versus 8, p=0.002) |
| Kanna [78] | Internal Medicine, USA, 2004 | Cohort | 81 | Among residents completing a reseach curriculum non-significant (p=0.053) increase proportion of those with at least one publication (21% vs 5%) |
| Khurana [88] | Neurology, India, 2010-2013 | Pre-post | Unclear (11 residents in department) | Implementation of a publication rotation associated with increased number of publications (27 vs 10) and total impact factor (117 vs 23; p=0.039) |
| Kichler [67] | General Surgery, USA, 2011-2013 | Pre-post | 14 | Implementation of a multi-faceted program increased publications (from 1 to 5) |
| Kohlert [35] | Otolaryngology, Canada, 1998-2013 | Cohort | 312 | Individuals who published during medical school were 5.85 times more likely to publish during residency (p<0.0001) |
| Konstantakos [60] | Orthopedic Surgery, USA, 2000-2009 | Pre-post | Unclear | Implementation of a mutlifaceted research team was associated with increased publications from 1 to 10 per year |
| Krueger [89] | Orthopedics, USA, 2007-2014 | Cohort | 3 training programs (100 residents per year in total) | Dedicated research time not associated with publication productivity (p=0.8) |
| Kurahara [70] | Pediatrics, USA, 1994-2007 | Pre-post | 135 residents | Implementation of multifaceted program increased publications from 2 to 26 (p<0.001) |
| Larsen [96] | Urology, USA, 2007-2019 | Pre-post | 30 | $1000 incentive for PubMed indexed publication resulted in increase from 2.44 to 6.33 average publications per year (p=0.0125) |
| Lee [82] | Urology, USA, 2002-2008 | Cohort | 543 | Increasing residency research time increased publications (p<0.001) |
| Lennon [58] | Family Medicine, USA, 2007-2012 | Pre-post | 30 residents | Increased research productivity following implementation of curriculum initiatives |
| Lepard [27] | Neurosurgery, USA, 2017-2018 | Survey | 102 (93%) of 110 programs | Publication productivity associated with amount of protected time (p<0.001) and journal club facilitators with biostatistics and epidemiology expertise (p=0.03) |
| Levitt [42] | Emergency Medicine, USA, 1996-1997 | Surveys | Program directors 107 of 116 (92%) and 51% (n=?) residents | No significant effect of mentor financial support, research award, or protected time on publications productivity |
| Levy [83] | Orthopedics, USA, 1997-2007 | Pre-post | 571 publications | After implementation of 80-hour work week limitations proportion of resident authorship in journal publications increased (12% to 18%; p=0.01) |
| Lohr [49] | General Surgery, USA, 1989-2004 | Pre-post | 137 publications | Since establishment of research teams publications increased by 13% from 60 to 77 |
| Lowe [76] | Psychosomatic and General Internal Medicine, Germany, 2005-2006 | Cohort | 37 | Registrants in 1-year research training program had nonsignificant difference in publications (p=0.3) |
| Manring [52] | Orthopedics, USA, 2006-2012 | Pre-post | ≈42 (6 per year) | Implementation of multifacteted program increased publication from 6 to 53 |
| McHenry [16] | Pediatrics, USA, 2014-2015 | Survey | 165 (63%) of 261 | Those interested in research were five times (p<0.05) more likely to be productive (publication or presentation at a national meeting) |
| Mezzacappa [66] | Psychiatry, USA, 2002-2011 | Pre-post | 52 | Implementation of a multifaceted program associated with increased publications (less than or equal to one to four papers) |
| Mills [43] | Family Medicine, USA, 1993 | Survey | 154 (86%) of 226 program directors | Programs with higher productivity had research mentor, faculty active in research, and larger programs |
| Millis [53] | Physical Medicine and Rehabilitation, USA, 1989-2003 | Pre-post | 142 | Implementation of multifaceted program associated with increases publications from 0.7 to 1.4 per resident per year (p=0.03) |
| Mlynarczyk [84] | Urology, USA, 2009-2013 | Cohort | 148 | h-indicies higher for trainees who took a dedicated 1 year research program (4.6 vs 8.1; p<0.001) |
| Morgan [48] | Radiation Oncology, USA, 2002-2007 | Cohort | 1,098 | Programs with more than 6 residents had higher rate of publications |
| Mutsaers [17] | Radiation Oncology, Canada, 2005-2016 | Cohort | 227 | Residents in later years of training and those enrolled in larger programs associated with higher publication rate |
| Oliver [54] | Emergency Medicine, USA, 2013-2018 | Pre-post | 32 | Implementation of a multifaceted program led to increased publications from 4 to 22 |
| Panchal [55] | Emergency Medicine, USA, 2002-2009 | Pre-post | 250 | Implementation of a multifaceted program led to increased publications from 6 to 16 |
| Papasavas [74] | General Surgery, USA, 2008-2012 | Pre-post | 33-35 residents per year | Implementation of a multifaceted research program did not increase publications (30 vs 32) |
| Roane [61] | Psychiatry, USA, 2002-2006 | Pre-post | 89 | Implementation of a multifaceted program increased publications from 1 to 15 per year |
| Robbins L [56] | Orthopedic Surgery, USA, 1998-2010 | Pre-post | 90 | Implementation of a multifaceted program led to increased publications from 0.5-2.0 pre to 9.3-10.8 post intervention per resident |
| Robbins MS [73] | Neurology, USA, 2005-2015 | Pre-post | 110 | Implementation of scholarly activity program non-significant increase in publications (0.75 to 1.00; p=0.36) |
| Roth [79] | Pediatrics, Canada, 2002-2005 | Pre-post | 43 | Implementation of a multifacteted research curriculum nonsignificant increase in publications (22% vs 15%; p=0.571) |
| Rothberg [59] | Internal Medicine, USA, 2001-2012 | Pre-post | 161 | Implementation of a multifaceted research program associated with increased publications from 3 to 39 (p<0.001) |
| Rowley [18] | Radiation Oncology, USA, 2015-2019 | Cohort | 909 | Males, larger programs, lack of doctorate degree associated with greater number of publications among those with at least one first author publication |
| Ruiz [62] | Internal Medicine, USA, 2006-2009 | Pre-post | 84 | Implementation of a multifaceted research program increased proportion of residents with a publication (32% vs. 7%; p=0.04) |
| Sabir [93] | Surgery, USA, 1996-2000 | Pre-post | ≈48 | Requirement for completion of 2 projects during residency increased publications from 2 to 12 per year |
| Sakai [65] | Anaesthesiology, USA, 2003-2011 | Mixed (Pre-post and cohort) | 119 in pre-post study and 258 in cohort study | Implementation of a multifaceted research program associated with increase in residents publishing (55.3% vs. 13.2%; p<0.0001) |
| Shah [25] | General Surgery, USA, 2007-2016 | Cohort | 36 | Completion of a degree during training associated with higher number of publications (median per year 3.8 vs 2.6; p=0.04) |
| Smith [24] | General Surgery, USA, 2001-2010 | Cohort | 69 | Completion of a degree during training associated with higher number of publications (10.3 vs. 5.3; p=0.001) and impact factor scores (32.3 vs 17.8; p=0.001) |
| Strauss [46] | Psychiatry, USA, 1977 | Survey | 155 (50%) of programs | University hospitals (2-fold), research rotation, separate research training track, and availability of a research mentor associated with publications (p<0.01 for each); requirement for a graduation report not associated with publications |
| Susarla [22] | Plastic Surgery, USA, 2009-2013 | Cohort | 78 abstracts with 50 (64%) published | Factors associated with publication was earlier year of training (p=0.003) and mentor rank as associate/full professor versus lower ranks (p=0.04) |
| Taniguchi [91] | Rehabilitation, USA, 1993 | Survey | 67 (89%) of 75 program directors | Requirement for research associated with nonsignificant increase in productivity (odds ratio 1.77; p=0.06). Mandatory research rotation or availability of elective time not associated with productivity |
| Taschanchai [38] | Pediatrics, Thailand, 1983-2012 | Cohort | 349 projects | Increased funding associated with number and impact factor of publications |
| Thiruthaneeswaran [37] | Radiation Oncology, Australia and New Zealand, 2005-2011 | Survey | 82 (71%) of 116 | Predominantly descriptive analysis only. Involvement of biostatistics support not associated with publication success |
| Thomas [92] | General Surgery, USA, 1965-1997 | Pre-post | 46 | Additional mandatory year of research increased publications from average to 2.67 from 0.94 per resident (p<0.001) |
| Tintara [94] | Obstetrics and Gynecology, Thailand, 1994-2003 | Pre-post | 759 | Implementation of a publication requirement did not increase overall publications |
| Torres [57] | Orthopedic surgery, USA, 2001-2012 | Pre-post | 51 | Implementation of a multifaceted research program was associated with increased (0.79 to 1.15 per resident; p=0.047) |
| Tsai [28] | Psychiatry, USA, 2000-2011 | Cohort | 48 | In an integrated program prior PhD published non-significant increased articles during training (2.6 vs. 0.8; p=0.9) |
| Verma [19] | Radiation Oncology, USA, 2014-2015 | Cohort | 334 | Males, larger programs, lack of doctorate degree associated with greater number of publications |
| Vernuccio [20] | Radiology, Europe, 2019 | Survey | 858 (unknown denominator) | Fewer females as compared to males (28% vs. 40%; p=0.001) published articles during residency |
| Villwock [90] | Otolaryngology, USA, 2016 | Survey | 38 (39%) of 98 programs | No factors found to be associated with publication productivity among programs surveyed |
| Vinci [21] | Pediatrics, USA, 2002 | Survey | 136 (82%) of 165 | Funding (63% vs 37%; p<0.0001) and project type (p=0.0002) associated with successful project (presented or published) |
| West [30] | Internal Medicine, USA, 2003-2009 | Cohort | 621 | Residents at the program published more articles (2.1 vs 0.6; p<0.001) than those who trained elsewhere; no difference in publication rates at program based on gender |
| Williams [85] | Orthopedic Surgery, USA, 2016 | Cohort | 1,690 trainees in 125 programs | Programs with protected research time had higher mean number of publications per resident (1.1 vs 0.6; p=0.02) |
| Winn [39] | Pediatrics, USA, 2003-2018 | Cohort | 248 | No difference in publication rate among those who received grants (9/71; 13%) or not (27/229; 12%; p=0.84) |
| Yumeen [40] | Plastic Surgery, Canada, 2000-2013 | Cohort | 126 | Higher rate of publication of basic science (9/14; 64%) as compared to clinical (42/113; 37%) projects presented at research day |
